# Fusion of HIV-1 Tat protein transduction domain to poly-lysine as a new DNA delivery tool

**DOI:** 10.1038/sj.bjc.6601680

**Published:** 2004-02-24

**Authors:** H Hashida, M Miyamoto, Y Cho, Y Hida, K Kato, T Kurokawa, S Okushiba, S Kondo, H Dosaka-Akita, H Katoh

**Affiliations:** 1 Department of Surgical Oncology, Division of Cancer Medicine, Hokkaido University Graduate School of Medicine, N-15, W-7, Kita-ku, Sapporo, Hokkaido 060-8638, Japan; 2 Department of Medical Oncology, Division of Cancer Medicine, Hokkaido University Graduate School of Medicine, Hokkaido, Japan

**Keywords:** HIV Tat protein, poly-lysine, DNA delivery

## Abstract

Effective gene therapy depends on the efficient transfer of therapeutic genes to target cells. None of the current technologies, however, satisfy all of the requirements necessary for gene therapy, because the plasma and nuclear membranes of mammalian cells are tight barriers against gene transfer using synthetic delivery systems. The protein transduction domain (PTD) of human immunodeficiency virus type 1 (HIV-1) Tat protein greatly facilitates protein transfer via membrane destabilisation. We synthesised polylysine peptides containing Tat PTD (TAT-pK), or other sequences, and investigated their potential as agents for gene transfer. The synthesised polypeptide TAT-pK retains DNA binding function and mediates delivery of a reporter gene to cultured cells. RGD motif binds with low affinity to alpha integrins which induce cell activation. Two control polypeptides, GGG-pK and RGD-pK, were synthesised and tested, but their gene transfer abilities were weaker than those of TAT-pK. TAT-pK-mediated gene transfer was enhanced in the presence of chloroquine or ammonium chloride, to a greater extent than that of cationic lipid-mediated gene transfer in most cancer cell lines tested. These data suggest that TAT-pK may be a potent candidate delivery vehicle that promotes gene transfer, dependent on the endocytic pathway. We conclude that the TAT-pK/DNA complex is useful as a minimal unit to package therapeutic genes and to transduce them into mammalian cells.

Gene therapy for cancer has been developed and a number of clinical therapeutic protocols are now being investigated. Vectors based on various viruses are useful for delivering therapeutic genes into primary cells *in vitro* and have also been applied in a number of gene therapy trials with humans. Viruses have some disadvantages as tools for medical application, however, with many elements of their biology yet to be elucidated. The utility of viral vectors for gene therapy is limited by DNA carrying capacity, difficulty in reliable and cost-effective manufacturing, and by immunogenicity and other safety concerns. One goal of cancer gene therapy is the development of gene delivery tools with lowered immunogenicity. While the construction of some viral vectors with reduced immunogenicity have been reported (Fisher *et al*, 1996; Haecker *et al*, 1996; Kochanek *et al*, 1996; Kumar-Singh and Chamberlain, 1996; Morral *et al*, 1999), preparation of these vectors is difficult because the virus is composed of several kinds of large molecules.

Two elements are necessary to efficiently express foreign genes in cells: passage of DNA across the cell membrane and transport into the nucleus (Colin *et al*, 2000). From this point of view, recombinant viral vectors have a great advantage by depending on their intrinsic machinery for infection. Basic peptides derived from human immunodeficiency virus type 1 (HIV-1) Tat protein and *Drosophila* Antennapedia protein have been reported to translocate through the cell membrane and to carry exogenous molecules into the cytoplasm and nucleus (Derossi *et al*, 1994,^1996^; Vivès *et al*, 1997; Nagahara *et al*, 1998; Schwartz *et al*, 1999; Dostman *et al*, 2000). HIV-1 Tat is an 86 amino-acid protein, and aa 47–57 of Tat (YGRKKRRQRRR) possess a high net positive charge at physiological pH, with nine of its 11 amino acids being either arginine or lysine. Fusion of several proteins and this 11 aa region of Tat protein enables the delivery of proteins into cells. Thus, this 11 aa region is considered a protein transduction domain (PTD). A 119-kDa protein, *β*-galactosidase, genetically fused to HIV-1 Tat PTD, was successfully carried into various mouse tissues, including the brain, following intraperitoneal injection (Nagahara *et al*, 1998).

Molecular conjugates of poly-lysine with natural or artificial ligands utilise the DNA-binding and -condensing properties of poly-lysine to mediate interaction with DNA (Wagner *et al*, 1991; Perales *et al*, 1994). Upon formation of a DNA-poly-lysine-ligand complex (polyplex (Felgner *et al*, 1997)), gene transfer is facilitated via receptor-mediated endocytosis.

In this study, we investigated the potential of a poly-lysine fused Tat PTD (TAT-pK) as a gene delivery agent. We demonstrate that TAT-pK combines with DNAs and efficiently transports them into several human cell lines.

## MATERIALS AND METHODS

### Reagents

Reagents were obtained from the following sources: chloroquine and ammonium chloride from WAKO Pure Chemical Industries, Ltd. (Osaka, Japan); 2,3-dioleyloxy-*N*-[2-(sperminecarboxamido)ethyl]-*N*′′*N*-dimethyl-1-propanaminium trifluoroacetate (DOSPA)/dioleoyl phosphatidylethanolamine (DOPE) (LipofectAMINE™) from Life Technologies, a division of Invitrogen (Rockville, MD, USA).

### Cell lines

The human embryonic kidney cell line HEK 293 was obtained from CLONTECH Laboratories. Human pancreatic carcinoma cell lines PCI10, PCI19, PCI35, and PCI43 were generously provided by Dr Yoshiki (Hokkaido University, Japan). Human oesophageal squamous cell carcinoma cell lines TE2, TE5, TE8, and TE13 were provided by Dr Nishihira (University of Tohoku, Japan). These cell lines were cultured in Dulbecco's modified Eagle's medium (DMEM; GIBCO, Rockville, MD, USA) with 2 mM L-glutamine, supplemented with 10% heat-inactivated foetal calf serum (FCS), at 5% CO2. Human lung carcinoma cell lines A549, RERF-LC-MS, and PC3 were obtained from the Japanese Cancer Research Resources Bank (Tokyo, Japan) and NCI-H226 was obtained from American Type Culture Collection (Manassas, VA, USA). They were maintained in RPMI medium (GIBCO) with 10% FCS.

### Polypeptides synthesis

Three pKs (TAT-pK, RGD-pK, GGG-pK) and TAT were supplied by Hokkaido System Science Co.,. Ltd. (Sapporo, Japan). They were chemically synthesised by solid-phase methods, using Fmoc (9-fluorenylmethyloxycarbonyl) with a Pioneer™ Peptide Synthesis System (Applied Biosystems, CA, USA). Primary structures of the polypeptides were shown in Table 1

**Table 1:** Primary structures of synthesised polypeptides

**Peptides**	**Sequences**
TAT-pK	NH_2_–YGRKKRRQRRR-GGG-KKKKKKKKKKKKKKK –COOH
RGD-pK	NH_2_–AIRGDTFATGAS-GGG-KKKKKKKKKKKKKKK –COOH
GGG-pK	NH_2_–GGG-KKKKKKKKKKKKKKK –COOH
TAT	NH_2_–YGRKKRRQRRR-GGG –COOH

.

### Plasmid DNAs

The expression plasmid for enhanced green fluorescent protein (EGFP) (CLONTECH Laboratories, CA, USA) was constructed by inserting the cDNA into pcDNA3.1+ to produce pcDNA-EGFP. The expression vector for firefly luciferase pGL3 was obtained from Promega Corp (Madison, WI, USA).

### Agarose gel electrophoresis of DNA-peptides complex

For the agarose gel electrophoresis assay, 0.5 *μ*g of DNA (lambda DNA/*Hind*III digest) and peptides were mixed and incubated for 10 min at room temperature. The samples were loaded on a 1% agarose gel containing 0.5 *μ*g ml^−1^ of ethidium bromide and run for 30 min at 100 V in 1 × TBE buffer.

### Transfection conditions

A standard protocol for gene transfer into cultured cells was followed. Cells were seeded at 2 × 10^4^–1 × 10^5^ cells well^−1^ in tissue culture plates, and cultured for 6 h. The cells were washed once with serum-free medium and incubated with medium containing DNA, DNA-peptides complex, or DNA complexed with cationic lipids (DOSPA/DOPE) for 8 h at 37°C. The cells were cultured for 48 h in medium with 10% FCS before assaying for the expression of reporter genes. DNAs were complexed with cationic lipids, according to the procedures recommended by the suppliers.

### Luciferase assay and UV microscopy

Luciferase activity was evaluated using the Luciferase Assay System (Promega) and relative light units (RLU) were measured with Mini Lumat LB 9506 (BERTHOLD, Germany). RLUs are shown as averages with standard deviations. GFP and FITC were detected with fluorescence microscopy (Olympus Optical Co. Ltd., Japan) using a GFPA cube. The cell nucleus was localised with fluorescence microscopy, using the fluorescent DNA binding dye, Hoechst 33342, and a WU cube (Olympus Optical Co. Ltd.). FITC labelled DNA fragments were prepared by phosphoramidite synthesis and purified by RP-HPLC purification.

### WST-8 assay

Cytotoxicity of peptides was investigated using WST-8 assay. HEK293 cells were seeded in 96-well tissue culture plate at a density of 2 × 10^4^ cells per well and incubated at 37°C for 72 h in fresh medium containing various peptides at a concentration of 10–320 *μ*g ml^−1^. After incubation, 10 *μ*l of 2-(2-methoxy-4-nitrophenyl)-3-(4-nitrophenyl)-5-(2,4-disulphophenyl)-2H-tetrazolium (WST-8; Wako Pure Chemical Industries, Ltd, Japan) were added to each well. After 4 h of incubation, the optical density was read on a microplate autoreader (SPECTRAmax® 190; Molecular Devices Corp, Sunnyvale, CA, USA) using a test wavelength of 490 nm and a reference wavelength of 620 nm.

### FACS analysis

Where indicated, 10 000 events were counted on a Becton Dickinson FACScan analyzer (Becton Dickinson, Franklin Lakes, NJ, USA) using a 15 mW air-cooled argon laser set at 488 nm, and recorded with a 530 nm emission filter in the FL1 emission channel. Cell populations are represented on a FACS histogram plotting FITC intensity on a logarithmic scale against cell number. Fluorescence intensity of cell populations is indicated by a shift to the right of the histogram plots of treated cells. Fluorescence enhancement was determined by obtaining the number of gated fluorescent events for untreated and treated cells.

## RESULTS

### DNA mobility shift analyses of pKs using agarose gel electrophorase

**Figure 1:**
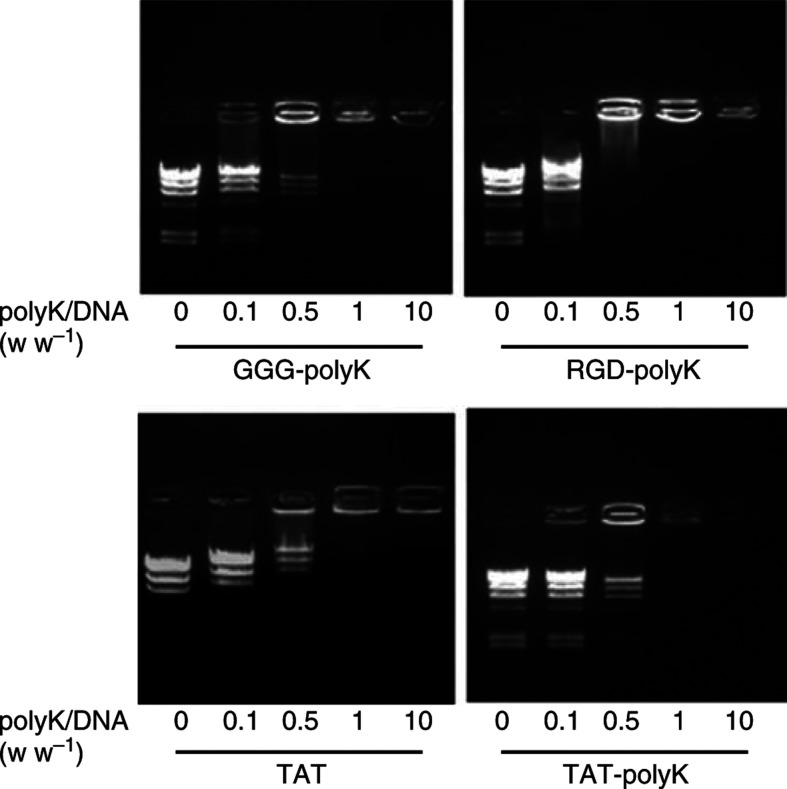
DNA mobility shift analyses of pKs. Synthesised pKs bind to plasmid DNA electrically. Three pKs were preincubated with 0.5 *μ*g of *λ*/*Hind*III DNA marker. The plasmid DNA was electrophoresed alone or after preincubation with a given concentration (0.05–5 *μ*g) of polypeptides. All plasmids were immobilised with pKs at a protein-to-DNA (w w^−1^) ratio of 1.

The pK tracts should impart DNA binding function to the fusion protein, by interacting with the negatively charged phosphate backbone of nucleic acids. To determine DNA binding ability, increasing concentrations of peptides were incubated with constant amounts of DNA marker (*λ*/*Hind*III), and the resulting effects on DNA mobility were analysed on agarose gels (Figure 1

). At concentrations where peptides completely bind and thus neutralise the DNA, it appears immobilised on the gel. The DNA was immobilised with peptides at a protein-to-DNA (w w^−1^) ratio of 1. These results show that peptides can bind DNA. Furthermore, excess amounts of peptides did not induce the DNA to migrate in the opposite direction from the positive electrode.

### Induction of EGFP and luciferase gene expressions by various peptides

**Figure 2:**
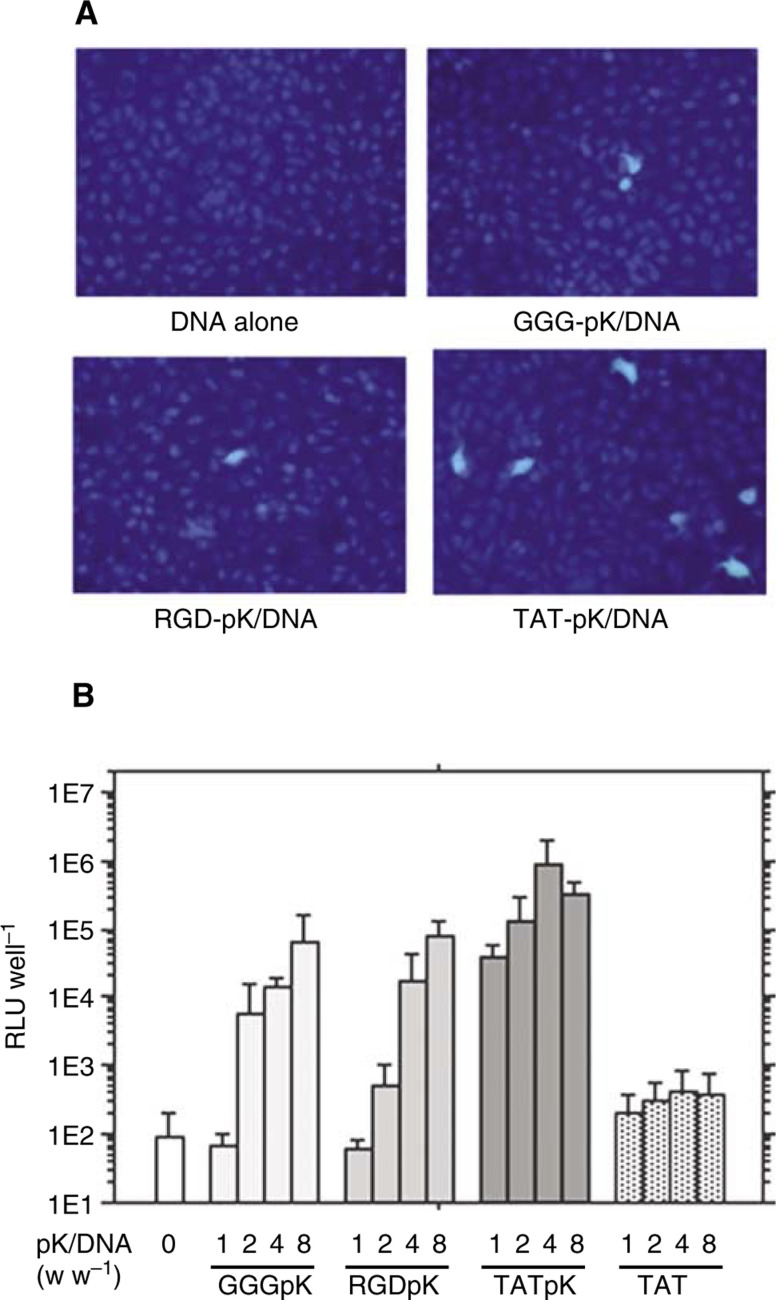
Induction of marker gene expression by various pKs. (**A**) *In situ* detection of pK-mediated GFP gene expression in HEK 293 cells. Cells were seeded in 12-well tissue culture plates at a density of 5 × 10^4^ cells well^−1^. The cells were treated with pK/pcDNA-EGFP complex (12 *μ*g of pKs and 3 *μ*g of DNA) as described under Materials and Methods. After 8 h, medium with 10% FCS was added, and the cells were grown for another 48 h before fluorescent microscopic observation. (**B**) Induction of peptide-mediated luciferase gene expression. Cells were seeded in six-well tissue culture plates at a density of 1 × 10^5^ cells well^−1^. The cells were treated with peptide/pGL3-promoter complex (5 *μ*g of DNA). After 8 h, medium with 10% FCS was added, and the cells were grown for another 48 h before they were harvested for analysis. Luciferase activity was evaluated using the Luciferase Assay System (Promega) and estimated in average relative light units (RLU) with standard deviations.

We examined the efficiency of peptide-mediated gene transfer by evaluating the expression of the complexed EGFP gene. Under standard transfection conditions, we detected strong EGFP expression in approximately 5% of HEK 293 cells treated with the TAT-pK/pcDNA-EGFP complex. In cells treated with the GGG-pK/ or RGD-pK/pcDNA-EGFP complex, however, EGFP expression was detected in less than 1% of cells (Figure 2A

). The number of cells expressing EGFP increased in proportion to the dose of pKs/DNA complex (figure not shown). In this study, the RGD-pK/DNA complex was also introduced into cells in a dose-dependent manner, but there was no difference in the transduction efficiency of RGD-pK and of GGG-pK. On the other hand, DNA complexed with TAT-pK was efficiently introduced, even at lower doses. The transduction efficiency of TAT peptide without a sequence of poly-lysine was lower than those of pKs (Figure 2B).

### Cell-binding activities evaluated by FACScan

**Figure 3:**
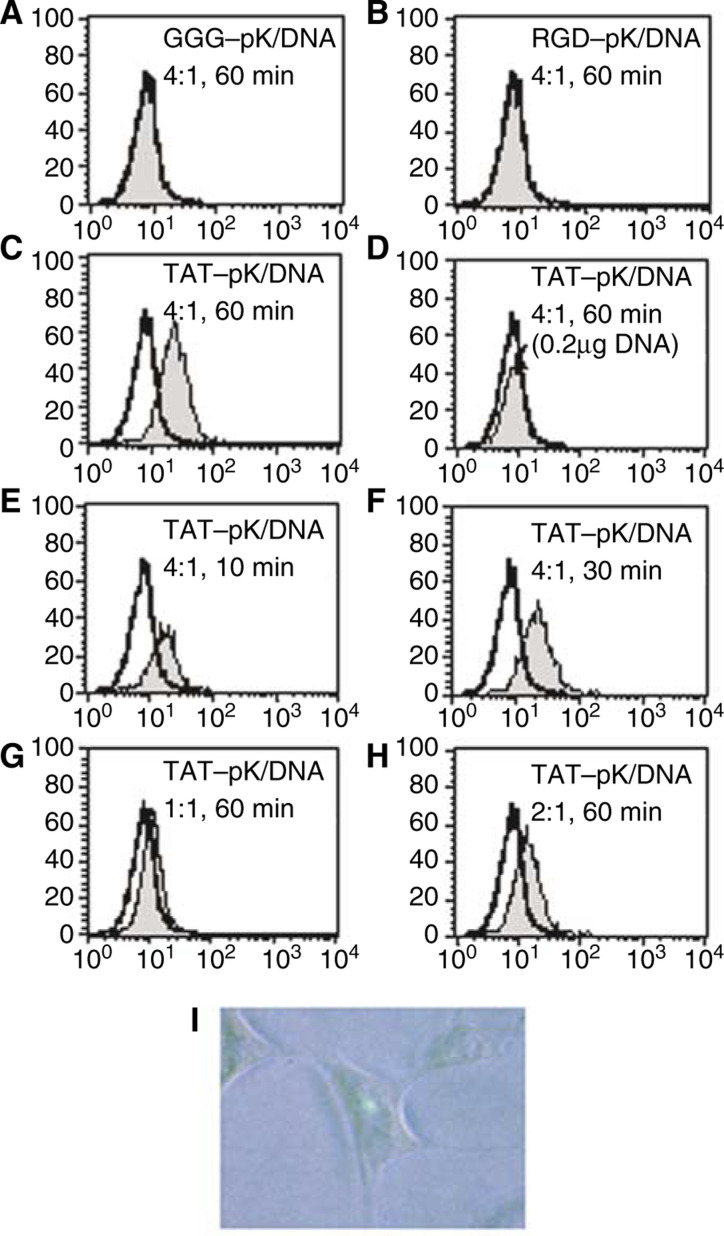
Cell binding activity of pK/DNA complex. HEK 293 cells were grown on 12-well dishes. Peptides and FITC-labelled-DNA were incubated together at the indicated ratios, at room temperature for 5 min in 1 ml of serum-free DMEM, and then added to cell monolayers. Cells were exposed to mixtures (2 *μ*g of DNA) for 60 min at 37°C, 5% CO_2_ (**A–C**). Cells exposed to mixtures containing 0.8 *μ*g of TAT-pK and 0.2 *μ*g of DNA for 60 min (**D**), 8μg of TAT-pK and 2 *μ*g of DNA for 10 min (**E**) and 30 min (**F**), indicated ratios of TAT-pK and 2 *μ*g of DNA for 60 min (**G**, **H**). *In situ* detection of TAT-pK/FITC-labelled-DNA complexes (**I**). Cells were exposed to mixtures containing 8 *μ*g of TAT-pK and 2 *μ*g of DNA for 2 h and FITC was detected with fluorescent microscopy as described under Materials and Methods.

To investigate the reason for the high efficiency of TAT-pK-mediated gene transfer, cell-binding activity of pK/DNA complexes using FITC-labelled DNA was assessed (Figure 3A-H

). There was scarcely any complex bound to cells with GGG-pK (Figure 3A) and RGD-pK (Figure 3B), and the amount of complex bound to cells did not increase, even 6 h after incubation (data not shown). TAT-pK/DNA complex, however, bound to almost all cells 60 min after incubation (Figure 3C). The difference in binding activity between TAT-pK and other pKs should be reflected in the DNA transduction efficiency. Interestingly, treatment with a small quantity of TAT-pK/DNA complex rarely led to cell binding (Figure 3D), and less than 0.1% of cells expressed EGFP when exposed to the equivalent quantity of TAT-pK/pcDNA-EGFP (data not shown). Most of the TAT-pK/DNA complex binds to the cells 10–30 min after incubation (Figure 3E and F). Tat-(48–60) has been reported to enter cells extremely rapidly, reaching the nucleus within 5 min (Futaki *et al*, 2001). Similarly, in our analysis, binding activity was observed immediately after incubation, and 1 h after incubation, FITC emission was detected in the nucleus (Figure 3I). No FITC emission was observed in cells exposed to the other pK/DNA complexes (data not shown). On the other hand, a four-fold excess of peptides, for neutralisation of the electrical charge, was required for high affinity (Figure 3G and H). A large number of peptides appear to require the complex to remain stably on the cell membrane or in the cytoplasm.

### Cytotoxicity of pKs evaluated by WST-8 assay

**Figure 4:**
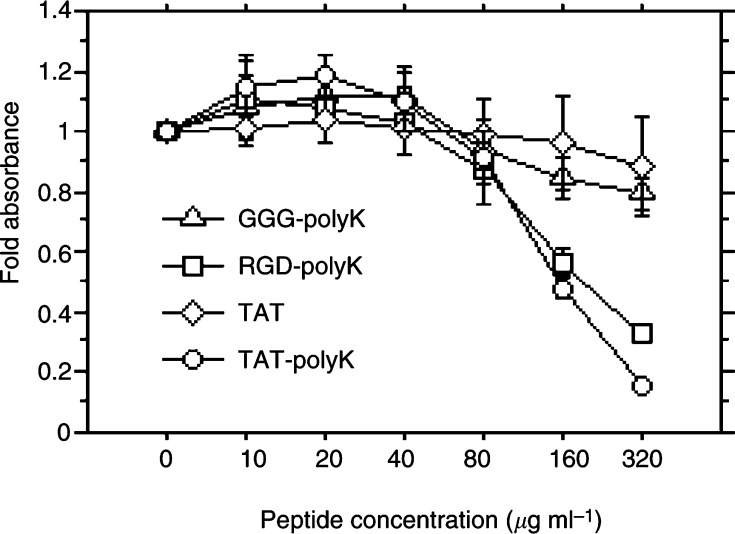
Cytotoxicity of polypeptides on HEK 293 cells. Cells were seeded into 96-well plates and incubated at 37°C for 72 h in fresh medium containing a given concentration of various polypeptides. Absorbance was measured by the WST-8 assay, as described under Materials and Methods. Each end point represents the mean±s.d.

The cytotoxicity of the three pKs was investigated after incubating 293 cells with peptide concentrations up to 320 *μ*g ml^−1^ for 72 h. The cytotoxicity of TAT-pK and of RGD-pK were almost equal, while that of GGG-pK was slight (Figure 4

).

### Characterisation of TAT-pK-mediated gene transfer in HEK 293 cells

**Figure 5:**
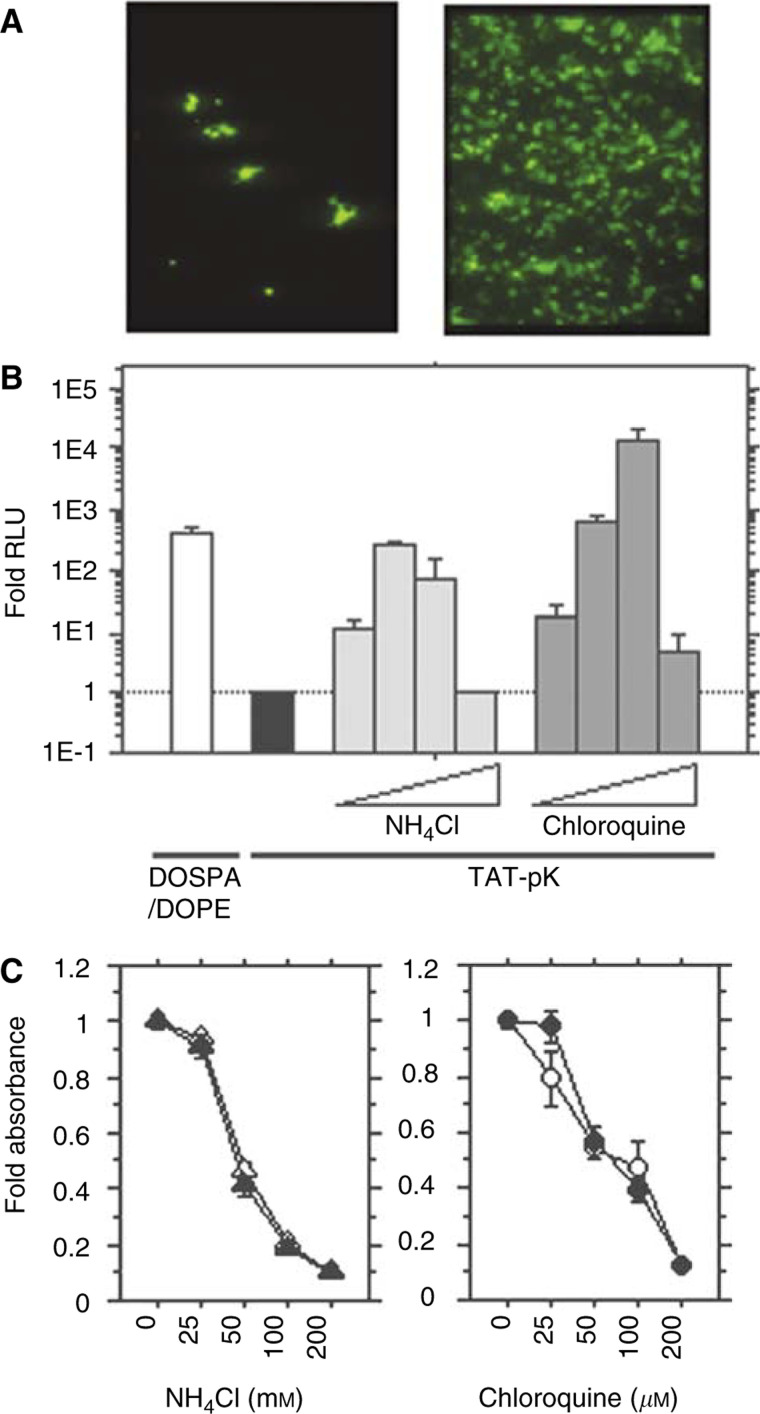
Characterisation of TAT-pK-mediated gene transfer in HEK 293 cells. (**A**) Enhancement of TAT-pK-mediated GFP gene expression in HEK 293 cells. Cells were seeded in 24-well tissue culture plates at a density of 2 × 10^4^ cells well^−1^. The cells were treated with 1 ml fresh medium containing TAT-pK/pcDNA-EGFP complex (4 *μ*g of peptide and 1 *μ*g of DNA) in the absence (left) or the presence of 100 *μ*M chloroquine (right). EGFP expression was detected with fluorescent microscopy as described under Materials and Methods. (**B**) Comparison of transfection activity of DOSPA/DOPE/DNA complex with TAT-pK/DNA complex and effects of ammonium chloride or chloroquine on TAT-pK-mediated gene transfer. HEK 293 cells (5 × 10^4^ well^−1^) were seeded into 12-well tissue culture plates. The cells were treated with DOSPA/DOPE/DNA complex (2 *μ*l of DOSPA/DOPA and 1 *μ*g of DNA, open bar), according to the procedures recommended by the suppliers, or with TAT-pK/DNA complex (4 *μ*g of peptide and 1 *μ*g of DNA) as described under Materials and Methods. The cells with TAT-pK/DNA complex were incubated for 48 h in the absence (filled bar) or presence (grey bars) of ammonium chloride (25, 50, 100, and 200 mM) or chloroquine (25, 50, 100, and 200 *μ*M). After incubation, cells were harvested and luciferase activity was evaluated. The luciferase activities were averaged from the results of duplicate experiments and are presented relative to the control value, indicated with the filled bar. (**C**) Cytotoxicity of ammonium chloride or chloroquine on HEK293 cells. Cells were seeded into 96-well plates and incubated at 37°C for 48 h in fresh medium containing a given concentration of ammonium chloride (left) or chloroquine (right) with (filled) or without (open) 20 *μ*g ml^−1^ TAT-pK. After incubation, absorbance was measured by the WST-8 assay, as described under Materials and Methods. Each end point represents the mean ± s.d.

TAT-pK-mediated EGFP expression was dramatically improved in the presence of 100 *μ*M chloroquine (Figure 5A

). Efficiency of TAT-pK-mediated luciferase expression was also elevated dose-dependently, but exposure to excess amounts of agent reduced the luciferase activity (Figure 5B). Moreover, as TAT-pK contains a GRKKR nuclear localisation signal within its sequence, we thought it would be advantageous for cellular gene expression. These functions are similar to those of viral vectors that actively bind to the cell surface via high-affinity ligands, and transport their DNA into the nucleus by endocytosis. Thus, TAT-pK/DNA complexes possess the necessary elements for transfection as a small particle, although the efficiency is lower than that of adenoviral vectors. When we compared the efficiency of TAT-pK-mediated gene transfer with that mediated by cationic lipids (DOSPA/DOPA), we found that TAT-pK can induce higher levels of luciferase activity in the presence of chloroquine, but not in its absence (Figure 5B). The cytotoxicity of ammonium chloride or chloroquine was investigated (Figure 5C). Both of ammonium chloride and chloroquine showed cytotoxicity at a higher concentration. There were no cytotoxic effects with TAT-pK.

### TAT-pK-mediated transduction efficiency in various human cancer cell lines

**Figure 6:**
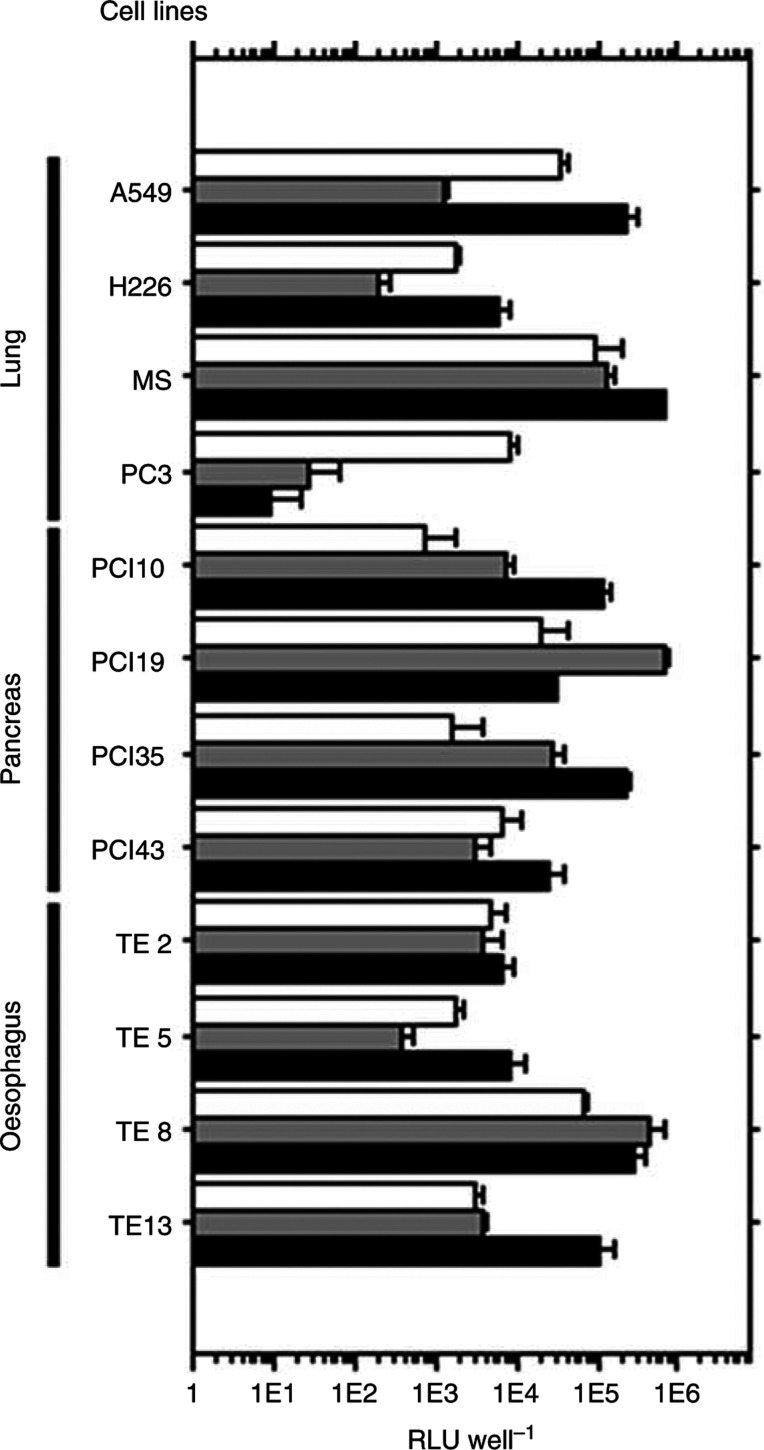
Transduction efficiency of DOSPA/DOPE or TAT-pK with chloroquine in various human cancer cell lines. Cells (5 × 10^4^) were incubated with 1 ml medium containing pGL3-promoter DNA (1 *μ*g) complexed with DOSPA/DOPA (open bars) or with TAT-pK in the presence of 50 *μ*M chloroquine (grey bars) or 100 *μ*M chloroquine (filled bars) and grown as described under Materials and Methods. After 48 h incubation, cells were harvested and luciferase activity was measured. Each end point represents the mean±s.d. RLU, relative light units.

To utilise TAT-pK for various purposes, we investigated the efficiency of TAT-pK-mediated luciferase gene transfer using several human cancer cell lines that tend to accept gene transfer with low efficiency (Figure 6

). The TAT-pK complex was successfully introduced into almost all cell lines at superior levels to DOSPA/DOPA, in the presence of chloroquine.

## DISCUSSION

In order to be utilised for gene therapy, gene delivery systems require convenience and safety. Even transporting a single DNA encoding a small protein needs a vector construct. Moreover, the vector has longer DNA and ‘the shell’ that wraps it. Because large vectors may have adverse effects, our aim was to construct the smallest possible unit permitting efficient and safe transfection of DNA into mammalian cells. Cationic polypeptides, such as poly-arginine and poly-lysine, have been reported to bind DNA, form complexes with DNA, and introduce themselves into cells (Wagner *et al*, 1991; Perales *et al*, 1994; Felgner *et al*, 1997; Futaki *et al*, 2001; Suzuki *et al*, 2002). Their efficiency for practical applications to gene therapy, however, remains untested. In the present study, we fused the Tat PTD to poly-lysine in order to improve the efficiency of DNA delivery.

As the pancreatic cancer cell line PCI35 has poor DNA transfection efficiency with cationic liposomes, we investigated modalities for efficiently transfecting pancreatic cancer cell lines. Initially, a recombinant Histidine6-tagged TAT-pK (H6-TAT-pK) construct was produced in *E. coli* strain BL21(DE3), carrying the pLysS plasmid (One Shot™; Invitrogen Corp., Carlsbad, CA, USA) to control leak-through expression and to allow subsequent cell lysis by freeze thawing. This construct was purified using a HiTrap™ column (Amersham Pharmacia Biotech., Buckinghamshire, UK) and the ÄKTA prime™ system (Amersham Pharmacia Biotech), and a standard protocol for protein production was followed. The H6-TAT-pK/DNA complex was successfully introduced into PCI35 cells and EGFP expression was detected. The efficiency of transfection, however, was not better than with lipofection (data not shown). Subsequently, in order to elucidate the mechanism for TAT-pK-mediated gene transfer and to improve the efficiency, we synthesised TAT-pK without the His6 tag, as well as two control polypeptides GGG-pK and RGD-pK. The adenoviral RGD (Arg-Gly-Asp) motif AIRGDTFATGAS was fused to pK to compare the transfection efficiency with that of TAT-pK, because interaction of the RGD motif in the adenoviral penton base with cell membrane integrins is required to induce or trigger endocytosis (Wickham *et al*, 1993).

We anticipated that the cytotoxicity of TAT-pK would be stronger than that of RGD-pK, but interestingly, this was not the case. Actually, as cells would not be exposed to high concentrations for a prolonged time, toxicity is not likely to be a serious concern. No toxicity was observed, even at the highest doses examined for DNA transduction.

Most nonviral vehicles deliver their genes passively, relying on uptake into vesicular compartments by endocytosis, thus we examined the effects of ammonium chloride or chloroquine on transduction. Ammonium chloride is a weak acidotropic base. Chloroquine elevates the pH of vesicular compartments (Cotten *et al*, 1990), and either stimulates or inhibits the efficiency of endocytosis-mediated gene transfer, depending on the delivery vehicle. We found that TAT-pK-mediated gene transfer is affected by either of these agents, suggesting that transduction relies on the endocytic pathway. These results are similar to past reports showing that gene transfer via receptor-mediated endocytosis (Cotten *et al*, 1990) or mediated by DEAE-dextran (Luthman and Magnusson, 1983) is markedly enhanced with endosomotrophic agents, such as chloroquine. However, our data contradict other studies on TAT-peptide-mediated protein transduction (Mann and Frankel, 1991; Derossi *et al*, 1996; Vivès *et al*, 1997; Elliott and O'Hare, 1997) and TAT-phage-mediated gene transfer (Eguchi *et al*, 2001) that do not depend on endosomotrophic reagents. The mechanism of action of Tat-(48–60) peptide and the full-length Tat protein may not be the same (Liu *et al*, 2000). Rather, TAT-pK-mediated gene transfer seems to share features of both systems, operating by both an energy-dependent endocytic pathway and an independent pathway. These dual mechanisms may account for the high efficiency of DNA transduction. In short, the Tat PTD anchors the TAT-pK/DNA complex to the cell surface within a few minutes by membrane destabilisation, and then the complex crosses the cell membrane by endocytosis.

The conditions suitable for gene transfer differed for each cell line. As PBS or RPMI medium reduce the efficiency of TAT-pK-mediated gene transfer (data not shown), we used sterilised water for diluting pKs and only used DMEM in transfection. Transduction efficiency of TAT-pK was easily influenced by several factors, such as pH or temperature of medium, preincubation period, and quantity of DNA (data not shown). Moreover, the fold absorptions were decreased at high concentrations of chloroquine because of its cytotoxicity.

Several Tat PTD fused proteins have been reported as potential therapeutic strategies for cancer (Mann and Frankel, 1991; Derossi *et al*, 1996; Elliott and O'Hare, 1997; Vivès *et al*, 1997), but the quantity of protein transduced into tissues would be lower than that from administration of vector DNA. Moreover, selectivity and transduction efficiency are very important factors in order to apply gene therapy for cancer patients. Although we have not achieved targeting transduction for cancer cells by using TAT-pK, it may be easily modified to target cancerous, but not normal cells, since TAT-pK is much smaller than the capsid proteins of viral vectors. Also immunogenicity by TAT-pK should be investigated, but we do not think it higher than that of viral vectors because of its size. We have started *in vivo* experiments to assess these factors and to improve them.

In conclusion, although there is need for further improvement, TAT-pK is a candidate for a new DNA transfection system. Many problems still exist in clinical trials using viral vector-mediated gene therapy, therefore the development of artificial viral vector systems is urgently needed. TAT-pK is likely a minimal unit to efficiently package therapeutic genes and transduce them into mammalian cells.
